# “Keep it a secret”: Leaked Documents Suggest Philip Morris International, and Its Japanese Affiliate, Continue to Exploit Science for Profit

**DOI:** 10.1093/ntr/ntae101

**Published:** 2024-06-27

**Authors:** Sophie Braznell, Louis Laurence, Iona Fitzpatrick, Anna B Gilmore

**Affiliations:** Tobacco Control Research Group, Department for Health, University of Bath, Bath, UK; Tobacco Control Research Group, Department for Health, University of Bath, Bath, UK; Tobacco Control Research Group, Department for Health, University of Bath, Bath, UK; Tobacco Control Research Group, Department for Health, University of Bath, Bath, UK; SPECTRUM (Shaping Public Health Policies to Reduce Inequalities and Harm) Consortium, Bath, UK

## Abstract

**Introduction:**

The tobacco industry has a long history of manipulating science to conceal the harms of its products. As part of its proclaimed transformation, the world’s largest tobacco company, Philip Morris International (PMI), states it conducts “*transparent science*.” This paper uses recently leaked documents from PMI and its Japanese affiliate, Philip Morris Japan (PMJ), to examine its contemporary scientific practices.

**Aims and Methods:**

Twenty-four documents dating 2012 through 2020 available from the Truth Tobacco Industry Documents Library were examined using Forster’s hermeneutic approach to analyzing corporate documentation. Thematic analysis using the Science for Profit Model was conducted to assess whether PMI and PMJ employed known corporate strategies to influence science in their interests.

**Results:**

PMJ contracted third-party external research organization, CMIC, to covertly fund a study on smoking cessation conducted by Kyoto University academics. No public record of PMJ’s funding or involvement in this study was found. PMJ paid life sciences consultancy, FTI-Innovations, ¥3 000 000 (approx. £20 000) a month between 2014 and 2019 to undertake extensive science-adjacent work, including building relationships with key scientific opinion leaders and using academic events to promote PMI’s science, products and messaging. FTI-Innovation’s work was hidden internally and externally. These activities resemble known strategies to influence the conduct, publication and reach of science, and conceal scientific activities.

**Conclusions:**

The documents reveal PMI and PMJ’s recent activities mirror past practices to manipulate science, undermining PMI’s proclaimed transformation. Tobacco industry scientific practices remain a threat to public health, highlighting the urgent need for reform to protect science from the tobacco industry’s vested interests.

**Implications:**

Japan is a key market for PMI, being a launch market for IQOS and having the highest heated tobacco product use globally. Our findings, in conjunction with other recent evidence, challenge PMI’s assertion that it is a source of credible science and cast doubt on the quality and ethical defensibility of its research, especially its studies conducted in Japan. This, in turn, brings into question the true public health impacts of its products. There is an urgent need to reform the way tobacco-related science is funded and conducted. Implementation of models through which research can be funded using the industry’s profits while minimizing its influence should be explored.

## Introduction

Science is essential to understanding and improving public health. Unfortunately, science is also used as a tool by corporate actors across diverse industries to conceal or create doubt about the harms of their products or manufacturing activities; to position their products as solutions to complex problems; and to legitimize their role in both science and policy.^1^ Corporate misuse of science is detrimental to public health as it delays and weakens policies, prevents litigation to protect consumers, and maximizes the use of potentially damaging products.^1^ These mechanisms ultimately serve to maximize corporate profits rather than primarily improve public health.^1^ Tobacco companies have a particularly well-documented history of scientific misconduct because of the release of internal industry documents which revealed they repeatedly prioritized their bottom line over the health of billions of people.^2^

Philip Morris International (PMI), the largest transnational tobacco company in the world,^3^ played a prominent role in the tobacco industry’s history of scientific misconduct and manipulation.^2^ In 2016, under threat from declining cigarette sales,^4^ PMI announced that it would be undergoing a “smoke-free” transformation, with the aim of replacing its cigarettes with its newer nicotine and tobacco products.^5^ Its flagship newer product brand is a heated tobacco product (HTP), IQOS, which it claims is less harmful than cigarettes.^6^

Scientific research is fundamental to PMIs claimed transformation and substantiating the harm reduction claims it makes about its newer products.^5^ PMI promotes its role in science to both the public and policy makers, proclaiming it conducts “*transparent science*.”^7^ Yet recent evidence contradicts this claim and raises questions about whether PMI has truly transformed.^8^ For example, the Foundation for a Smoke-Free World (FSFW) claims to be independent but is solely funded by PMI and publishes PMI-favorable research,^6,9^ mirroring the long-standing tobacco industry practice of using scientific third parties to promote products and corporate messaging.^10^ Moreover, journalist investigations and academic reviews of PMIs science have raised serious concerns over the quality and ethical standing of PMIs clinical research.^11,12^

In this context, a small sample of recently leaked documents relating to the scientific activities of PMI and its Japanese affiliate, Philip Morris Japan (PMJ), provide limited but unique insight into PMIs contemporary scientific practices. Japan is of particular interest because it has the highest prevalence of HTP use in any country worldwide.^13^ PMI therefore often uses Japan as the location for its studies on HTPs, including those submitted to regulatory bodies in the U.S. and EU,^14,15^ and publicly promotes Japan as “*an example of successful harm reduction to other countries*.”^16^

The aims of this study were to examine these new documents in order to gain insight into PMIs contemporary research practices and use of science; assess whether PMI continues to employ known strategies to influence science; and discuss the implications of its practices for wider tobacco research.

## Materials and Methods

We examined a set of internal PMI and PMJ documents from an ex-PMJ employee, published in the University of California San Francisco’s Truth Tobacco Industry Documents library. This set included 24 documents, comprising contracts, E-mails, invoices, presentations and reports relating to PMI and PMJs scientific activities between 2012 and 2020. Ten of the documents were in English and 13 in Japanese, for which we used the English translations (provided by the library).

We conducted a detailed examination of the documents, drawing on a hermeneutic approach to company document analysis,^17^ which has been used previously to examine documents on the FSFW^18^ and tobacco industry.^19,20^ This approach aims to provide an in-depth understanding of the meaning and significance of documents through repeated reading; contextualizing findings within the time, place, and settings of the documents; and triangulating with other sources. The documents were read and their meta-data (eg file number, name, date, subject, etc.) indexed in Microsoft Excel. The documents were then contextualized and triangulated to check the reliability and validity of their contents. Names and positions of individuals identified were confirmed via LinkedIn profiles, university Web sites and ORCID. Literature databases (Google Scholar and PubMed), clinical trial databases (ClinicalTrials.gov and UMIN.ac.uk), and academics’ research profiles were used to identify evidence on studies referenced in the documents, research interests of named academics, and connections between parties. PMI Web sites (pmi.com and pmiscience.com) and Web sites of other companies named in the documents were searched for further evidence of activities identified in the documents. Conference Web sites were searched to confirm the attendance of individuals named in the documents. Other contextual sources were identified through news media and Google searches using the names of individuals, companies, events, and other keywords referenced in the documents. Where possible, we conducted searches using a Japanese IP address via a VPN and used Japanese terms taken directly from the documents, such as names of organizations and events. Webpages in Japanese were translated to English using Google Chrome’s built-in translate functionality.

Documents were repeatedly re-read in the context of other sources and authors regularly met to discuss interpretations. A timeline of events was developed, and key overarching activities were identified. Once familiar with the documents and broader context, three authors (SB, LL, and IF) independently conducted a thematic analysis. Similar to a recent study on FSFW documents,^18^ analysis coding was based on the Science for Profit Model (SPM)—a typology of industry influence on science developed using historical evidence on the activities undertaken by tobacco and other industries to influence science and the use of science.^1^ The model comprises three increasingly specific levels of strategies: macro, meso, and micro. We deductively analyzed the documents using the micro-level strategies (shown in Table 1). Once each author had completed independent analysis, all authors met to resolve any disagreements and agree on a final list of strategies identified within the documents.

**Table 1.** The Science for Profit Typology. Micro-Level Strategies Were Used as Codes in the Thematic Analysis and Are Shown in the Context of the Macro-Level Strategies

A. Influence the conduct and publication of science to skew evidence bases in the industry’s favor
1.1 Fund and undertake “safe” research which distracts attention from industry harms, frames industry and industry products as part of the “solution,” and promotes interventions that minimize damage to product sales
1.2 Commission lawyers and public relations firms to manage research programs to ensure research is “safe”
1.3 Fund and undertake research to identify or demonstrate public perceptions
2.1 Covertly undertake “risky” research so that it can be hidden or abandoned
2.2 Prevent “risky” industry research from being undertaken
3.1 Control the design and analysis of industry-funded primary studies
3.2 Control the design and analysis of industry-funded evidence syntheses
4.1 Shape external (eg governmental) organizations’ research priorities through access, funding, and political power
4.2 Attempt to block the funding of potentially unfavorable independent research
4.3 Deliberately obstruct independent data collection
5.1 Maximize the presence of industry-funded publications in the peer-reviewed literature
5.2 Fund or create journals to have influence over what is published
5.3 Create publications which emulate peer-reviewed/quality science
6.1 Control the way in which unfavorable industry science is reported within publications
6.2 Suppress publication of unfavorable science

B. Influence the interpretation of science to undermine unfavorable science and create a distorted picture of the evidence base
7.1 Develop criteria for the conduct and interpretation of science (including determining scientific “proof”) with the intention that these can be used to undermine unfavorable science
7.2 Adopt the concepts of “junk science” and “sound science” for use in undermining science and promoting industry science
7.3 Fund and coordinate public relations campaigns to promote these industry-friendly criteria and concepts to key stakeholders
8.1 Obtain raw data underlying unfavorable research by enabling data access legislation, and pressuring or litigating against scientists
8.2 Re-analyze independent data including that squired in these ways to refute unfavorable findings
9.1 Attack the methods of unfavorable science (tailoring the criteria depending on the nature of the science to be attacked)
9.2 Label unfavorable science “junk”
9.3 Misrepresent single pieces of evidence
9.4 Misrepresent whole evidence bases
9.5 Misrepresent expert consensus
10.1 Monitor the opposition in order to weaken it
10.2 attack individual scientists and whole cohorts of researchers
10.3 Remove individual scientists from positions of power
10.4 Attack organizations that create and disseminate science

C. Influence the reach of science to create an “echo chamber” for the industry’s scientific messaging
11.1 Prevent research from being undertaken in countries where corporations are vulnerable to litigation based on legal advice
11.2 Limit internal communication to mask industry knowledge of harms based on legal advice
11.3 Store scientific documents in ways which would prevent their discovery based on legal advice
11.4 Use “proprietary information” claims when pressed by courts to release industry evidence
11.5 Attempt to embed mechanisms in trade and investment treaties which prevent access to industry science
11.6 Silence plaintiffs using secret payments
12.1 Create front groups to amplify industry-friendly scientific messages
12.2 Fund third parties in order to amplify or shape their scientific stances
12.3 Recruit, fund, and train individuals to be trusted scientific voices for the industry
12.4 Strategically create and fund a multitude of voices to manufacture a picture of scientific consensus
12.5 Build industry coalitions (both within an industry and with allied industries) to demonstrate support for industry-friendly scientific stances
13.1 Fund, produce and disseminate lobbying materials that summarize science in industry-favorable ways
13.2 Fund, produce and disseminate textbooks, letters to the editor, practice guidelines and other educational or academic materials
13.3 Fund, produce and disseminate “easily digestible” materials such as factsheets, newsletters, and product information materials
13.4 Obtain and disseminate reprints of industry-favorable scientific publications
14.1 Fund, organize and speak at “educational events” for key stakeholders
14.2 Access policy makers through meetings and hearings
14.3 Infiltrate decision-making contexts in order to ensure industry-friendly scientific stances are heard
14.4 Train and use industry representatives to meet with health professionals
15.1 Create favorable media content
15.2 Fund media outlets in order to influence what is disseminated
15.3 Co-opt journalists through media training and conference funding
15.4 Design events intended to maximize media coverage
15.5 Demand media coverage of industry scientific arguments in the name of “balance”

D. Create industry-friendly policy making environments which shape the use of science in policy decision making in the industry’s favor
16.1 Attempt to implement industry-friendly standards of evidence which set a high evidential bar within regulatory decision making
16.2 Utilize such standards in attempts to undermine the use of unfavorable science in regulatory decision making
17.1 Secure the implementation and use of mandatory regulatory tools such as business impact assessments which increase reliance on industry data and prioritize evidence on economic impacts
17.2 Secure and utilize changes to upstream policy making architecture which embed the industry’s right to participate in policy making processes
17.3 Secure reductions in evidence requirements for some regulatory decision making to maximize the use of industry-favorable evidence

E. Manufacture trust in the industry and its scientific messaging
18.1 Ensure and normalize the industry’s presence in academic settings in attempts to gain trust and scientific credibility within academia
18.2 Promote the industry’s overt links with expert individuals and organizations to manufacture a picture of industry credibility more broadly
18.3 Inflate the credibility of industry science, scientists, and scientific perspectives
19.1 Conceal industry funding of science, production of science and recruitment of scientists
19.2 Conceal industry dissemination of science and scientific messages
19.3 Conceal industry attempts to shape ways in which science is used in policy making

All monetary figures used throughout the text are also provided in Pounds Sterling using average conversion rates from ExchangeRates.org.uk at the time of payment.

## Results

### Overview of Events

**Figure 1.**
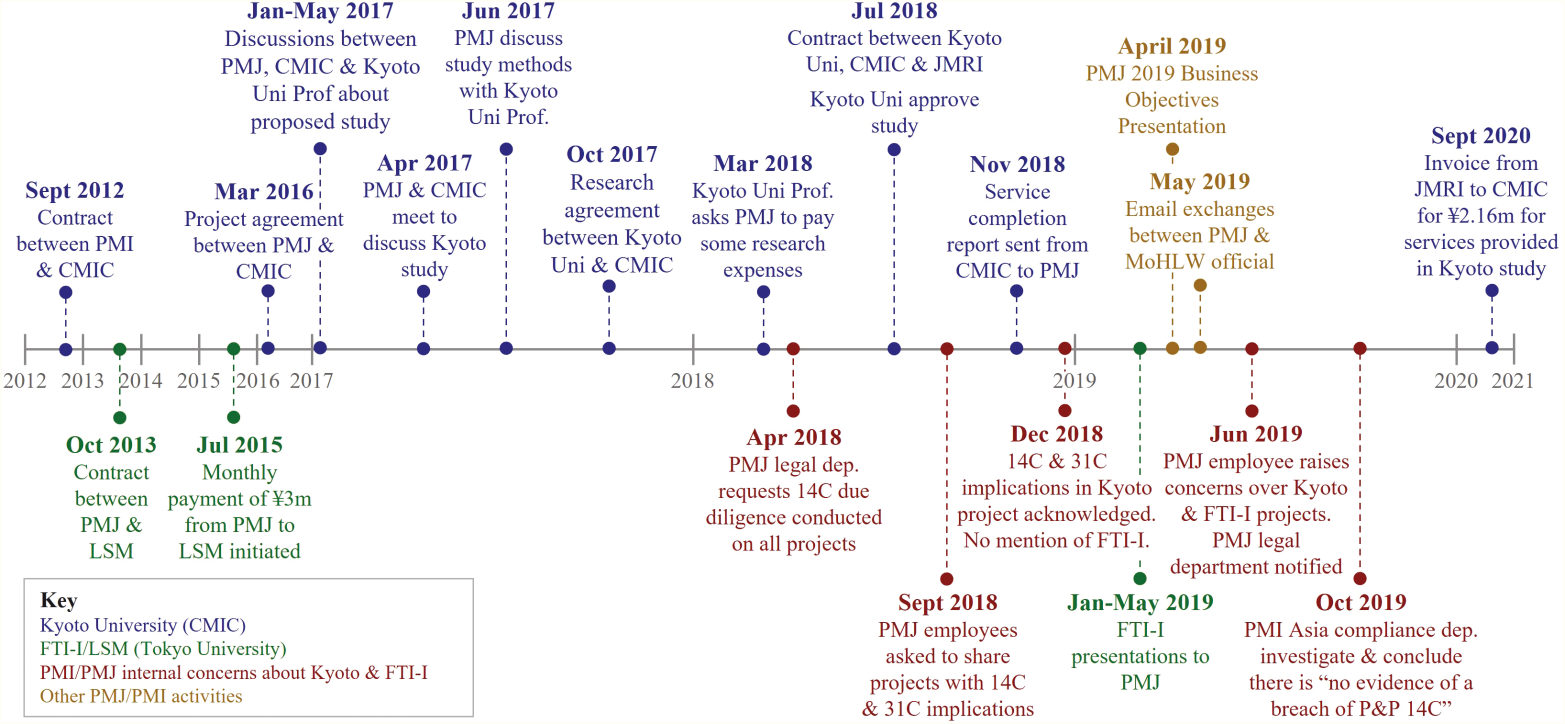
Timeline of events identified from the PMI and PMJ internal documents; color coded to four over-arching activities (see key). Abbreviations: PMI and PMJ: Philip Morris International/Japan; LSM: Life Science Management Inc; FTI-I: FTI Innovations Inc (formally LSM); CMIC: CMIC Inc; JMRI: Japan Medical Research Institute Co., Ltd; MoHLW: Japanese Ministry of Health, Labor and Welfare.

Figure 1 provides a timeline summarizing key activities evidenced in the documents that took place between 2012 and 2020. We grouped these into four overarching activities identified in our initial examination: PMJ’s collaboration with Kyoto University; PMJs collaboration with FTI Innovations, Inc, (FTI-I); internal PMJ and PMI concerns around these collaborations; and other PMI and PMJ activities identified in the documents.

Below we describe PMI and PMJs activities under each of these themes in more detail. The SPM strategies mirrored in these activities are signposted throughout the text and summarized in Table 2.

**Table 2.** Summary of PMJ and PMI Activities and Which Strategies They Mirror From the Science for Profit Model. Activities Are Grouped Under Macro-Level Strategies. Numbers in the Righthand Column Denote Micro-Level Strategies

Activity	SPM strategy mirrored
A. Influence the conduct and publication of science to skew evidence bases in industry favor	
FTI-I conducted research for PMI, including research on KOLs to target, conferences to attend, and Japanese regulations relevant to PMI products.	1.3^a^; 2.1
PMJ employees in charge of Kyoto University and FTI-I collaborations limited internal communication with the PMJ legal department and did not conduct appropriate due diligence in a timely manner.	2.1
PMJ indirectly funded an “*epidemiological study*” by Kyoto University academics through CMIC. All parties are aware PMI was funding the study.	2.1
FTI-I provided support and advice on PMI’s research, including practical support for a “*medical economic model*,” “*advice on test protocol, ethics and COI*,” “*supporting opportunities for discussions with overseas researchers*,” and “*introduction of outsourcing companies in the medical field*.”	3.1; 3.2
CMIC provided “*scientific support*” to PMJ and PMI on its clinical trials	3.1
PMJ were involved in the Kyoto University study concept and design.	3.1
PMI shared U.S. regulatory decision on IQOS directly with a Japanese MoHLW official.	4.1
C. Influence the reach of science to create an “echo chamber” for the industry’s scientific messaging	
PMJ employees in charge of Kyoto University and FTI-I collaborations limited internal communication with the PMJ legal department and did not conduct appropriate due diligence in a timely manner.	11.2
FTI-I organized symposiums at the University of Tokyo, which PMI employees attended.	12.3; 14.1; 18.1
PMI used FTI-I to disseminate its science and other favorable evidence via workshops, conferences and discussions with stakeholders.	12.3; 12.5; 14.1
PMJ used FTI-I to “*maintain and expand relationships with KOLs*” in scientific, medical, pharmaceutical, insurance and public health communities, as well as “*Building relationships with dentistry, insurance and pharmacists*.”	12.4; 12.5; 14.1; 14.4
PMJ used FTI-I to promote PMI’s science, products and corporate messaging through medical and scientific communities and KOLs.	12.2; 12.5; 14.1
PMI 2019 business objectives seek to disseminate its science and scientific messaging, eg “*expand 3rd party scientific endorsement*” and use “*Japan KSOLS present Japan HNB success story at key international events*”	12.1; 12.2; 12.3; 12.4; 12.5; 14.3; 15.1
PMI shared the U.S. regulatory decision on IQOS directly with a Japanese MoHLW official.	14.3
E. Manufacture trust in the industry and its scientific messaging	
PMI and PMJ planned to send an employee to the University of Tokyo as a visiting researcher, organized via FTI-I.	18.1
FTI-I conducted research for PMI, including research on KOLs to target, conferences to attend, and Japanese regulations relevant to PMI products.	19.1
PMJ indirectly funded an “*epidemiological study*” by Kyoto University academics through CMIC. All parties are aware PMI was funding the study.	19.1
PMJ employees were told to keep collaboration between FTI-I and PMJ “*a secret*.”	19.1

^a^Adapted from 1.3 (amendments in italics): Fund and undertake research to identify or demonstrate *scientific, medical & public health communities’* perceptions.

Abbreviations: SPM: Science for Profit Model; PMJ: Philip Morris Japan; PMI: Philip Morris International; FTI-I: FTI Innovations Inc; CMIC: CMIC Inc; K[S]OLs: key [scientific] opinion leaders; HNB: heat-not-burn; THR: tobacco harm reduction; COI: conflict of interest; MoHLW: Ministry of Health, Labor, and Welfare.

### PMJs Collaboration With CMIC and Kyoto University

From 2012, PMI contracted CMIC Holdings Co. Ltd.^21^ – Japan’s largest contract research organization that offers support in pharmaceutical development, from pre-clinical studies through to post-approval marketing.^22^ According to the leaked contracts, CMIC provided PMI and PMJ with “*consultancy services in support of* [PMJ’s] *scientific engagement activities*”^23^ (strategy 3.1). This included translation services; consulting on potential studies with key opinion leaders (KOLs) in Japan, communicating with doctors or clinical sites involved in PMI studies, data management and analyses; planning and supporting epidemiological research; and supporting the conduct of clinical trials in Japan on “*NGPs*.”^21,23–28^ We believe “*NGPs*” stands for next-generation products, a term commonly used by PMI and other tobacco companies to describe newer nicotine and tobacco products, like IQOS.^29^

Examination of publicly available materials revealed CMIC managed PMIs published clinical trials on IQOS in Japan, some of which were the subject of a 2017 Reuters exposé.^11^ In the exposé, employees of PMI and contracted companies involved in the trials described issues, such as collecting participants’ urine samples without signed consent forms, submission of urine samples exceeding the volume of human capability, concerns over proper screening of participants, and inadequate training of investigators.^11^ Despite this, registration of an ongoing clinical trial on IQOS in Japan,^30^ suggests PMI continues to contract CMIC to manage its clinical trials.

**Figure 2.**
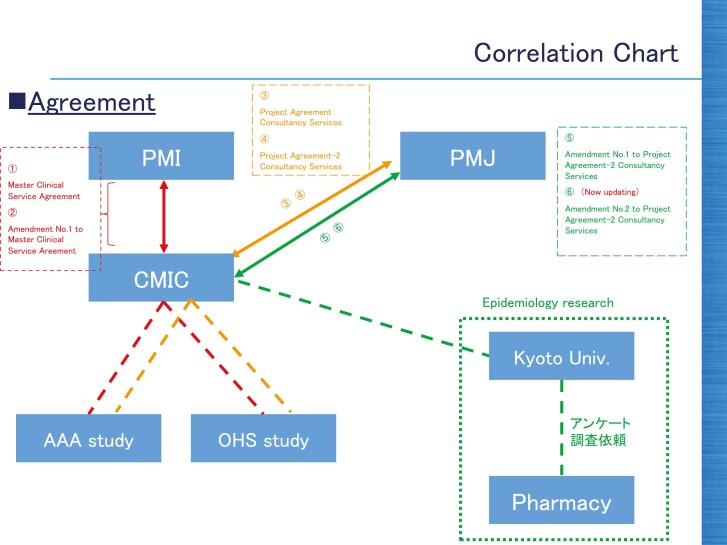
Summary of the relationships and contracts (numbers 1–6) between PMI, PMJ, CMIC, Kyoto University, and related studies.^28^ We believe the “AAA” and “OHS” studies refer to PMI clinical trials: P1-AAA-02-JP^84^ and P1-OHS-01-JP.^53^ The Japanese text appears to reference the research questionnaires for the study on cessation. Abbreviations: PMI: Philip Morris International; PMJ: Philip Morris Japan; Univ: University.

Between 2017 and 2019 PMJ funded a study by Kyoto University academics through CMIC. The study involved using medical databases to analyze the prevalence, adherence and usefulness of smoking cessation aids in Japan, plus conducting surveys in pharmacies to collect data on trends in smoking and health effects before and after cessation.^31^ Although not directly investigating IQOS, PMJ decided the study was worthwhile as it could provide the company with “*beneficial information for public health*.”^32^ The relationship between PMI and PMJ, CMIC, and Kyoto University was summarized in a diagram,^28^ shown in Figure 2.

The leaked documents suggest there was no contract between PMI and PMJ and the Kyoto University academics. Instead, E-mails between CMIC, PMJ, and the Kyoto academics suggest the study funding was attributed to CMIC, despite all three parties being aware PMJ was providing the study funds^32–35^ (strategies 2.1 and 19.1). PMJ were also in direct discussions regarding the design of the study (strategy 3.1). The study concept sheet (attached to a leaked E-mail but not accessible) was shared by Kyoto academics with PMJ employees, who said they had reviewed it and suggested amendments.^32^ The same E-mail thread indicated the academics were to meet with CMIC “*to discuss data collection and management, etc.*” and PMJ employees wanted to discuss study “*test details and questions*” with the academics directly.^32^

In 2018, CMIC and Kyoto University jointly contracted Japan Medical Research Institutes Co. Ltd. (Japan Medical),^36^ a consultancy subsidiary of Japan’s largest pharmacy company, Nihon Chouzai Co. Ltd.^37^ The contract stated CMIC was to pay Japan Medical 4 million Yen (£27 200) to conduct the pharmacy surveys with individuals using smoking cessation products who visit Nihon Chouzai pharmacies between July 2018 and June 2019.^36^ This suggests the “*Pharmacy*” in Figure 2 referred to Nihon Chouzai.

In November 2018, CMIC sent PMI a “*Service Completion Report*,”^31^ and in September 2020, Japan Medical sent an invoice to CMIC, totaling 2.16 million Yen (£15 768).^38^ No study findings were described in the documents. Despite the documents indicating the study was completed, we could not identify any publications, references to the study or declarations of the academics’ relationship with PMJ in literature databases, university Web sites or the academics’ profiles. Two of the academics involved in this study have since published a paper on heated tobacco products in which they declared previous funding from CMIC but failed to mention PMI and PMJ.^39^

### PMJ’s Collaboration With FTI-I and LSM

In 2013, PMI initiated a contract with FTI-I, known as Life Science Management, Inc, (LSM) at the time.^40^ FTI-I is a life science consulting firm based in Japan, whose Representative Director (the senior executive in charge) is also a Professor at the University of Tokyo.^41^ Between 2014 and 2019, PMJ paid FTI-I 3 million yen a month (roughly £19–22 000) to support PMJ’s scientific activities.^42^ The contract between FTI-I and PMJ has not been publicly acknowledged by either party.

**Figure 3.**
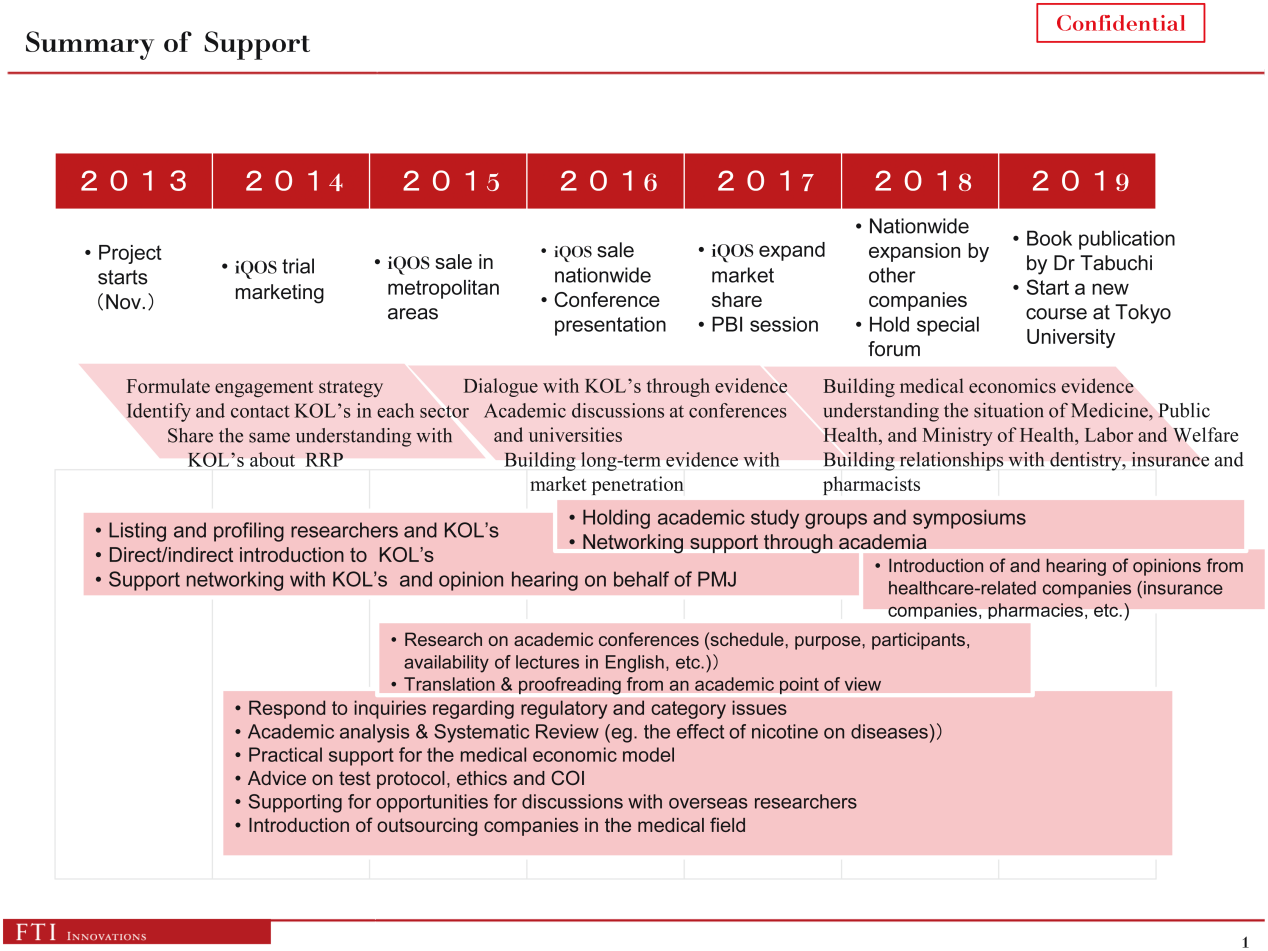
FTI-Is summary of the support it has provided PMJ 2013–2019 (slide 4 of FTI-I presentation “Discussion Material”).^43^ Abbreviations: KOL: key opinion leader; PMJ: Philip Morris Japan; RRP: reduced risk products; COI: conflict of interest. It is unclear what “PBI” stands for.

In a 2019 presentation to PMJ, FTI-I provided a summary of its work for PMJ (Figure 3, text in pink boxes).^43^ This included identifying relevant academic conferences and whether tobacco industry-affiliated content is permitted^43,44^; detailing regulatory processes, like the Pharmaceutical and Medical Device Act in Japan^44^; conducting academic analyses and systematic reviews^43^; interviewing KOLs in the medical community (adapted strategy 1.3)^45^; and profiling KOLs from scientific, medical and public health communities.^43–45^ We found no publicly available materials on these aspects of FTI-I’s work.

FTI-I also advised on and supported PMJs own scientific research, including “*practical support for the medical economic model*,” “*advice on test protocol, ethics and COI*” (conflict of interests), “*supporting opportunities for discussions with overseas researchers*,” and “*introduction of outsourcing companies in the medical field*”^43^ (strategies 3.1 and 3.2). As there is no further reference to these activities within the documents, it is unclear whether they pertain to any of PMIs published studies.

FTI-I disseminated PMIs scientific research through workshops, study groups, conferences, and discussions with KOLs and stakeholders, sometimes organized by FTI-I itself (strategies 12.3, 12.5, 14.1, and 14.4).^42–45^ For example, it organized a symposium at the University of Tokyo, attended by PMI employees,^42,45^ ran a special lecture at the University of Tokyo on HTPs at which a PMJ employee was a speaker,^44^ and identified relevant conferences at which PMI could present its research (strategy 14.1).^42,44,45^ Altogether this helps to ensure PMI and PMJ’s presence in academic settings, building support and credibility among relevant academic communities (strategy 18.1).

There were also plans, orchestrated by FTI-I, to send a PMI employee as a visiting researcher to the University of Tokyo,^42,45,46^ where FTI-Is Representative Director is a professor.^41^ The exact purpose and duration of this proposed visit was unclear, though FTI-I stated it would be to “*facilitate* [PMJ’s] *activities*”^45^ and PMJ listed it as a target under its goal to “*expand fiscal differentiation for HNB*”^46^ (HNB stands for heat-not-burn, an alternative term for HTPs).^29^ We found no external evidence indicating a PMJ employee had been sent to the University of Tokyo.

FTI-I sought to “*maintain and expand relationships with KOLs*”^45^ in scientific, medical and public health communities by profiling potential KOLs, arranging introductions and collaborations, and building dialogue channels^42–45^ (strategy 12.4). Indeed, in one 2019 FTI-I presentation, six medical and health professionals are listed as examples of KOLs FTI-I had already introduced to PMJ. One of these has gone on to promote harm reduction, HTPs, IQOS, and PMI’s science to the media^47–49^ and at several academic events.^0–999^

It also sought to build relationships with other sectors, such as insurance, pharmaceutical industries and the dentistry community (strategy 12.5).^43^ In 2019 FTI-I specifically wanted to gain access, build connections, seek collaborations, and obtain endorsements from the Japanese dental community, and had already spoken with some dentists.^45^ At the time, PMI had an ongoing clinical trial in Japan investigating the effects of IQOS on oral health and periodontitis treatment.^54^

### PMI and PMJ’s Internal Concerns

Throughout 2018, it appears PMJ employees attempted to limit internal communication around the FTI-I project (strategy 11.2). Content relating to the proposed visiting researcher from PMI and information on KOLs in public health was removed from one 2019 FTI-I presentation.^45^ Additionally, the documents suggest the full extent of the FTI-I project was hidden from PMJs own legal department. In 2018, the legal department requested updates from PMJ employees on any projects relevant to PMIs policies around contracted third parties that interact with government officials on PMJs behalf; conduct or disseminate policy, product- or science-related messaging of interest to PMI; or participate or organize events where policy or science relating to PMI products is discussed.^40^ PMJ employees notified the legal department of the CMIC and Kyoto University project but neglected to mention the FTI-I project.^42^ Moreover, the documents reveal PMJ had not conducted appropriate due diligence on the FTI-I project since 2013.^40^

In June 2019, a PMJ employee E-mailed both the company’s president^56^ and PMJs Director for People & Culture Japan^57^ expressing concerns over the FTI-I project, describing it as “*a potentially risky issue for PMJ reputation*.”^42^ The employee claimed the FTI-I director had urged PMJ employees not to talk about the collaboration between FTI-I and PMJ externally and “*to keep it a secret*”^42^ (strategy 19.1). The employee highlights the aforementioned issues of FTI-I in the context of PMI’s policies on contracted third parties—the absent notification to the legal department and the lack of due diligence. Following this, the legal department was notified of the project.^40^ A subsequent internal investigation conducted by PMI concluded there was no breach of PMI’s policies.^58,59^ There was no evidence among the documents to suggest any updated due diligence was conducted or that PMJs contract with FTI-I had ceased.

### Other PMJ and PMI Activities

In May 2019, PMJ employees E-mailed the Tobacco Free Initiative Officer for the Japanese Ministry of Health, Labor and Welfare (MHLW)^60^ to explain IQOS had been granted permission to be marketed in the U.S.^61^ The Officer congratulated PMJ on the “*great news*” and asked to be kept informed of PMI’s subsequent application to market IQOS using harm reduction claims in the U.S. The decision in the U.S. was largely predicated on scientific research provided by PMI in its application, including its clinical and post-market studies from Japan.^14^ We found no external evidence linking the Officer and PMI and PMJ. However, sharing this PMI-favorable regulatory decision from the U.S. with regulators from Japan may be an attempt to shape the latter’s policy and research priorities (strategy 4.1) or infiltrate decision-making contexts to ensure the inclusion of scientific stances that are beneficial to the company (strategy 14.3). In the year following this E-mail exchange, the Officer published (as first author) two studies on HTP emissions,^62,63^ both of which support the use of HTPs indoors and were subsequently used by PMI to assert independent science claims its HTPs are less harmful than cigarettes.^64^ Both studies cite funding from the MHLW and all authors declared no conflicts of interest. Further suggesting attempts to shape policy and research, the documents reveal FTI-I had also introduced PMJ to the Director of the Japanese National Institute of Public Health,^45^ which provides the MHLW with science to support public health policy development and implementation.^65^

In a 2019 presentation, PMJ described its business goals as growing its “*reduced risk*” product market, increasing IQOS use amongst current and new consumers, building societal support, expanding differentiated regulation and accelerating conversion to its “*reduced risk*” products.^46^ It is unclear if “*new consumers*” refers to current nonsmokers. To achieve these, PMI set out a variety of objectives (Box 1) relating to dissemination and amplification of its harm reduction science and messaging across politics and academia (strategies 12.1–12.4), engaging governments to ensure its stances are heard (strategy 14.3), and maximizing media coverage of favorable science (strategy 15.1).^46^ The leaked documents and publicly available materials suggest work was carried out to meet these objectives, including engaging with 15 prefectural governments,^46^ attempting to send a PMJ employee to the University of Tokyo as a visiting researcher,^42,45,46^ leveraging the Japanese National Fire Agency report^66,67^ and Australian Government application.^68^ In line with its objectives PMJ engaged with the Asian Population and Development Association (APDA), including sponsoring an APDA seminar in November 2020 in which the Secretary General of the Japan Parliamentarians Federation for Population spoke in favor of incorporating harm reduction in health policy.^69^ Further, PMI employees and scientists funded by PMI via the FSFW^70,71^ presented at the Global Tobacco & Nicotine Forum 2019^72^ and the Global Forum on Nicotine 2019. The aforementioned KOL introduced to PMJ by FTI-I^43^ presented on the status of tobacco harm reduction in Japan at the same Global Forum on Nicotine^51^ and on “*Policy Interventions for Differentiated Products*” at the 2019 Africa Harm Reduction Forum.^50^

Box 1. **Examples of PMJs 2019 business objectives.^46^**

- “Engage 30 prefectural governments” and “Engage 43 local governments” (slide 4).
- “PMI presentation to MOF, MOH, politicians, academics” (we understand MOF and MOH to mean Ministry of Health and Ministry of Finance, respectively; slide 4).
- “PMI and PMJ personnel invited as visiting scholar to Tokyo University School of Life Science” (slide 4).
- “Secure domestic political support and make progress towards inclusion of harm reduction at regional/international parliamentary events” (slide 5).
- “Expand third party scientific endorsement for tobacco harm reduction” (slide 5).
- “Leverage [National Fire Agency] report for engagement and media” and “Engage [Asian Population and Development Association] to introduce harm reduction concept” (slide 5).
- “Japan KSOLS present Japan HNB success story at key international events” (KSOLS: key scientific opinion leaders), including the Global Forum on Nicotine, Africa Tobacco Harm Reduction Forum and Global Tobacco & Nicotine Forum (slide 5).
- Implement “communications to boost societal awareness and support for THR and Smoke-Free Japan” (slide 6).
- “PMJ presence at Olympic venue” and “Cooperate with House of Switzerland” (House of Switzerland is a Swiss Government managed organization which showcases Swiss news, brands and products;^85^ slide 6).

## Discussion

In this study, we used hermeneutic and thematic analyses to examine a small set of leaked documents to investigate the contemporary scientific activities of the world’s largest transnational tobacco company in one of its key markets, Japan. We found evidence that PMJ funded a study through a third party (CMIC) on smoking cessation in Japan by university academics, who have since neglected to declare their relationship with PMJ in a subsequent publication.^39^ CMIC also managed PMI’s clinical research, even after concerns had been raised both publicly by Reuters^11^ and internally by a PMJ employee. We found further evidence that PMJ paid FTI-I to undertake a range of science-adjacent work, including providing PMJ with access to key opinion leaders and events through which it promoted its desired rhetoric. Directly and through FTI-I, we also found evidence of potentially unethical interactions between PMJ and significant individuals involved in public health policy and research. Our analysis showed these activities mirror strategies previously used by the tobacco industry to influence science in the industry’s interest. Namely, influencing the conduct and publication of science (SPM macro strategy A), influencing the reach of science and scientific messaging (strategy C), and concealing these activities (strategy E).

While our analysis can only provide a relatively narrow insight into PMIs contemporary scientific activities, our findings need to be considered alongside other evidence. This includes evidence that PMI funds a seemingly independent scientific group, the FSFW,^9^ published in journals with editorial board members that have ties to PMI,^73^ promoted and attended conferences run by predatory conference organizers,^74^ attacked independent science,^6^ and trained media through tours of its scientific facilities.^6^ Furthermore, a recent systematic review noted numerous inadequacies in the conduct and reporting of PMIs clinical trials on its HTPs, all of which were judged to be at high risk of bias.^12^

PMI and PMJ’s activities undermine not only the company’s assertions that it conducts “[r]*igorous, robust, and transparent science*,”^7^ but also wider public health. PMI has used its own science to appeal for more industry-favorable regulation^14,15^ and make harm reduction claims that have contributed to increased consumption of PMI’s HTPs, notably IQOS.^75–77^ Of greater pertinence, is that some of PMI’s most significant clinical and post-market studies were conducted in Japan, having been included in past submissions to governments,^14,15^ and PMI has used seemingly independent science to further substantiate claims that its HTPs are beneficial to public health.^16,78^ However, our findings raise concerns about the integrity of this evidence base and, by extension, the true harms of PMIs products.

PMJs covert funding of research further confuses the evidence base by hindering our ability to ascertain which academics are truly independent. We identified instances of academics presenting at conferences or publishing papers without declaring their connections to PMI, despite existing journal policies against accepting tobacco industry research^79^ and previous recommendations to Japanese medical societies to improve conflict of interest policies.^80^ Requirements for authors to accurately disclose not only direct tobacco industry funding but also indirect funding should be enforced by conference organizers and publishers. Cases of dubious disclosure should be pursued and consequences clearly stated.

The world’s largest cigarette manufacturers, including PMI, generate huge profits—substantially more than Coca-Cola, Pepsico, Nestle, Mondelez, FedEx, General Mills, Starbucks, Heineken, and Carlsberg combined^81^ and possess significant research and development resources.^82^ Thus, companies like PMI have the potential to meaningfully contribute to science. Unfortunately, evidence suggests the vested interests of these companies continue to subvert this potential. There is therefore an urgent need to develop funding models whereby the tobacco industry’s vast profits can be used to fund scientific research without influence or bias. As posited by Cohen et al.,^83^ such models should seek to ensure transparent and independent funding mechanisms and governance, use competitive funding processes, protect against selective results reporting and conflicts of interest, set research agendas independently of sponsors, minimize the industry’s PR gains from research, and be feasible for implementation.

Governments and regulatory bodies considering permitting the sale or regulation of PMI’s newer products, especially IQOS, should be aware of the company’s activities in Japan and carefully scrutinize research from the region. It is imperative national and international Governments drive research reform. This could involve exploring proactive measures to prevent corporate research malpractice (including the aforementioned funding models), exercising oversight, ensuring proper investigation and levying penalties against activities like those revealed in these PMI and PMJ documents.

The strengths and limitations of this study lie in the dossier of leaked documents used in our analyses. On the one hand, they provide unique insight into PMIs contemporary research practices, and we are the first to examine this particular set of documents. We conducted extensive searches and investigatory research for external sources to triangulate the information and activities described in the documents. The use of previously proprietary documents, especially ones from such recent times, also provides a level of insight into the actions and intentions of companies like PMI that can otherwise be difficult to discern using historical documentation or publicly available data sources. On the other hand, this study is restricted by the scope of the documents. The full extent of PMI’s activities, both in Japan and globally, cannot be determined from this one analysis. The uncovering of PMIs scientific activities in Japan is particularly significant because it is a key market for the company and often the location of its clinical and post-market studies.

## Conclusions

In conclusion, building on continuing emerging evidence, the leaked documents reveal PMI continues to employ strategies to influence science at the expense of public health. PMIs activities in Japan undermine the company’s proclaimed transformation, questioning the veracity of the evidence based on its products and, in turn, casting doubt on their true harms. Continued skepticism of its activities and published research is certainly justified. Regardless of the company’s intentions, PMIs activities highlight the urgent need to reform the way the tobacco industry funds and conducts research and other scientific activities. Funding models that protect the transparency and integrity of tobacco-related science should be explored and implemented.

## Supplementary Material

Supplementary material is available at *Nicotine and Tobacco Research* online.

ntae101_suppl_Supplementary_Material

## Data Availability

The data underlying this article are available in the article and on the University of California San Francisco’s Truth Tobacco Industry Documents library.

## References

[1] Legg T , Hatchard J, Gilmore AB. The science for profit model—how and why corporations influence science and the use of science in policy and practice. PLoS One. 2021;16(6):e0253272. 34161371 10.1371/journal.pone.0253272 PMC8221522

[2] Bero LA. Tobacco industry manipulation of research. Public Health Rep. 2005;120(2):200–208. 15842123 10.1177/003335490512000215 PMC1497700

[3] Forbes. *Leading tobacco companies worldwide in 2021, based on market value (in billion U.S. dollars). Statista*. 2022. https://www.statista.com/statistics/942132/leading-10-tobacco-companies-worldwide-based-on-net-sales/. Accessed November 28, 2022.

[4] Gilmore AB , Branston JR. Philip Morris International: The Beginning of the End? *Expose Tobacco.* 2020. https://exposetobacco.org/news/pmi-agm/. Accessed February 9, 2023.

[5] Philip Morris International. *Our Transformation*. PMI. 2022. https://www.pmi.com/our-transformation/our-interactive-transformation. Accessed November 28, 2022.

[6] Karen Evans-Reeves. Addiction at Any Cost. Philip Morris International Uncovered. STOP. https://exposetobacco.org/wp-content/uploads/STOP_Report_Addiction-At-Any-Cost.pdf; Published 2020. Accessed November 28, 2022.

[7] Philip Morris International. *The research and evidence to date on smoke-free products*. PMI Science. 2023. https://www.pmiscience.com/en/research/. Accessed November 28, 2023.

[8] Edwards R , Hoek J, Karreman N, Gilmore A. Evaluating tobacco industry ‘transformation’: a proposed rubric and analysis. Tob Control. 2022;31(2):313–321. 35241605 10.1136/tobaccocontrol-2021-056687

[9] Legg T , Legendre M, Gilmore AB. Paying lip service to publication ethics: scientific publishing practices and the Foundation for a Smoke-Free World. Tob Control. 2021;30(e1):e65–e72. 33911028 10.1136/tobaccocontrol-2020-056003 PMC8606453

[10] Ong EK , Glantz SA. Constructing “sound science” and “good epidemiology”: tobacco, lawyers, and public relations firms. Am J Public Health. 2001;91(11):1749–1757. 11684593 10.2105/ajph.91.11.1749 PMC1446868

[11] Lasseter T , Bansal P, Wilson T, Wilson A, Kalra A. Special Report - Scientists describe problems in Philip Morris e-cigarette experiments. Reuters. 2017. https://www.reuters.com/article/idUSKBN1EE1G7/. Accessed November 28, 2022.

[12] Braznell S , Van Den Akker A, Metcalfe C, et al. Critical appraisal of interventional clinical trials assessing heated tobacco products: a systematic review. *Tob Control.* 2024;33:383–394. 36347620 10.1136/tc-2022-057522 PMC11041615

[13] Donahue TS. Heating Up. Tobacco Reporter. 2021. https://tobaccoreporter.com/2021/07/01/heating-up-2/. Accessed February 9, 2023.

[14] U.S. Food & Drug Administration. *Philip Morris Products S.A. Modified Risk Tobacco Product (MRTP) Applications*. FDA. 2022. https://www.fda.gov/tobacco-products/advertising-and-promotion/philip-morris-products-sa-modified-risk-tobacco-product-mrtp-applications. Accessed February 9, 2023.

[15] Philip Morris International. *Tobacco Heating System (THS)'s Scientific Dossier submitted in line with EU’s Tobacco Products Directive*. PMI Science. 2019. https://www.pmiscience.com/en/smoke-free/tobacco-regulation/eu-tobacco-products-directive/. Accessed February 9, 2023.

[16] Philip Morris International. *Harm reduction: A focus on Japan*. PMI Science. 2022. https://www.pmiscience.com/content/dam/pmiscience/en/pdfs/scientific-update-magazin/pmi-scientific-update-issue-16r-japan.pdf. Accessed February 9, 2023.

[17] Forster NS. The analysis of company documentation. In: Cassell C, Symon G, eds. Qualitative Methods in Organizational Research: A Practical Guide. London, UK: SagePublications; 1997.

[18] Legg T , Clift B, Gilmore AB. Document analysis of the Foundation for a Smoke-Free World’s scientific outputs and activities: a case study in contemporary tobacco industry agnogenesis. Tob Control. 2023:tc–2022. 10.1136/tc-2022-057667 PMC11228203 37137700

[19] Peeters S , Gilmore AB. Transnational tobacco company interests in smokeless tobacco in Europe: analysis of internal industry documents and contemporary industry materials. PLoS Med. 2013;10(9):e1001506. 24058299 10.1371/journal.pmed.1001506 PMC3769209

[20] Peeters S , Gilmore AB. Understanding the emergence of the tobacco industry’s use of the term tobacco harm reduction in order to inform public health policy. Tob Control. 2015;24(2):182–189. 24457543 10.1136/tobaccocontrol-2013-051502 PMC4345518

[21] Philip Morris Products SA. *Master Clinical Services Agreement*. 2012. Philip Morris International. https://www.industrydocuments.ucsf.edu/docs/ppcv0284. Accessed June 14, 2024.

[22] CMIC HOLDINGS Co., LTD. *Contract Research Organization, Your End-to-End Solutions Provider*. CMIC. 2022. https://en.cmicgroup.com/. Accessed November 28, 2022.

[23] Philip Morris Japan. *Project Agreement - Consultancy Services*. 2016. Philip Morris International. https://www.industrydocuments.ucsf.edu/docs/rpcv0284. Accessed June 14, 2024.

[24] Philip Morris International. *Amendment No. 1 to Master Clinical Services Agreement*. 2014. Philip Morris International. https://www.industrydocuments.ucsf.edu/docs/qpcv0284. Accessed June 14, 2024.

[25] Philip Morris Japan. *Project Agreement-2. Consultancy Services*. 2016. Philip Morris International. https://www.industrydocuments.ucsf.edu/docs/spcv0284. Accessed June 14, 2024.

[26] Philip Morris Japan. *Amendment No. 1 to Project Agreement-2. Consultancy Services*. 2018. Philip Morris International. https://www.industrydocuments.ucsf.edu/docs/tpcv0284. Accessed June 14, 2024.

[27] Philip Morris Japan. *Amendment No. 2 to Project Agreement-2. Consultancy Services*. 2019. Philip Morris International. https://www.industrydocuments.ucsf.edu/docs/zpcv0284. Accessed June 14, 2024.

[28] Iida T. *Kyoto Contract Correlations* . 2014. Philip Morris International. https://www.industrydocuments.ucsf.edu/docs/gqcv0284. Accessed June 14, 2024.

[29] Tobacco Tactics. *Tobacco Industry Product Terminology*. Tobacco Tactics. 2023. https://tobaccotactics.org/article/tobacco-industry-product-terminology/. Accessed February 9, 2023.

[30] Philip Morris Products SA. *A multicenter, open-label, three-parallel group study to assess the velocity of abdominal aortic aneurysm (AAA) dilatation in adult smokers randomized to either cigarette smoking or IQOS use and compared with abstinent patients. Controlled study*. UMIN. 2023. https://center6.umin.ac.jp/cgi-open-bin/ctr/ctr_view.cgi?recptno=R000040452. Accessed February 9, 2023.

[31] Kondo Y. *Service Completion Report - Study on smoking cessation with medical/drug database by conducting research in pharmacies nationwide* . 2018. Philip Morris International. https://www.industrydocuments.ucsf.edu/docs/jqcv0284. Accessed June 14, 2024.

[32] Iida T. *RE: Concept Sheet on Smoking Cessation Research (Yoshida, Kyoto University)* . 2017. Philip Morris International. https://www.industrydocuments.ucsf.edu/docs/hpcv0284. Accessed June 14, 2024.

[33] Kawakami K. *RE: Progress on Smoking Cessation Research (Yoshida, Kyoto University)* . 2018. Philip Morris International. https://www.industrydocuments.ucsf.edu/docs/jpcv0284. Accessed June 14, 2024.

[34] Kawakami K. *RE: Thank you for the meeting (CMIC Kondo)* . 2017. Philip Morris International. https://www.industrydocuments.ucsf.edu/docs/lpcv0284. Accessed June 14, 2024.

[35] Iida T. *RE: Philip Morris Epidemiological study on smoking cessation (Kondo, CMIC)* . 18 May 2017. Philip Morris International. https://www.industrydocuments.ucsf.edu/docs/ypcv0284. Accessed 14th June 2024.

[36] Unknown. *Service Agreement (Kyoto University, CMIC Co. Ltd., Japan Medical Research Institute Co., Ltd.)*. 2018. Philip Morris International. https://www.industrydocuments.ucsf.edu/docs/sycv0284. Accessed June 14, 2024.

[37] Nihon Chouzai. *Nihon Chouzai Group. Nihon Chouzai*. 2023. https://www.nicho.co.jp/en/profile/group/. Accessed February 9, 2023.

[38] Japan Medical Research Institute Co. Invoice No. G3006-T6409. 2020. Philip Morris International. https://www.industrydocuments.ucsf.edu/docs/hqcv0284. Accessed June 14, 2024.

[39] Takenobu K , Yoshida S, Katanoda K, Kawakami K, Tabuchi T. Impact of workplace smoke-free policy on secondhand smoke exposure from cigarettes and exposure to secondhand heated tobacco product aerosol during COVID-19 pandemic in Japan: the JACSIS 2020 study. BMJ Open. 2022;12(3):e056891. 10.1136/bmjopen-2021-056891 PMC8935009 35304398

[40] Iida T. *Cooperation with Prof. Kimura in University of Tokyo* . 2019. Philip Morris International. https://www.industrydocuments.ucsf.edu/docs/tycv0284. Accessed June 14, 2024.

[41] FTI Innovations. *About us*. FTI-I. 2022. https://fti-innovations.wixsite.com/website/about. Accessed November 28, 2022.

[42] Unknown. RE: *An issue of potential reputational risk of PMJ*. 2019. Philip Morris International. https://www.industrydocuments.ucsf.edu/docs/fpcv0284. Accessed June 14, 2024.

[43] FTI Innovations Co. *Discussion Material*. 2019. Philip Morris International. https://www.industrydocuments.ucsf.edu/docs/zycv0284. Accessed June 14, 2024.

[44] FTI Innovations Co. *Report of FY2018*. 2018. Philip Morris International. https://www.industrydocuments.ucsf.edu/docs/xpcv0284. Accessed June 14, 2024.

[45] FTI Innovations Co. *Discussion Material*. 2019. Philip Morris International. https://www.industrydocuments.ucsf.edu/docs/gpcv0284. Accessed June 14, 2024.

[46] Philip Morris Japan. *PMJ 2019 Business Objectives - Q1-Q3 Targets*. 2019. Philip Morris International. https://www.industrydocuments.ucsf.edu/docs/mpcv0284. Accessed June 14, 2024.

[47] Tobacco Reporter. Hiroya Kumamaru. *Tobacco Reporter*. 2020. https://tobaccoreporter.com/2020/10/24/hiroya-kumamaru/. Accessed May 5, 2023.

[48] NZZ Content Solutions. Convincing expert colleagues of the concept of harm reduction. *Neue Zürcher Zeitung*. 2020. https://www.nzz.ch/themen-dossiers/magazine-inside-innovation-for-philip-morris-4-convincing-expert-colleagues-of-the-concept-of-harm-reduction-ld.1567651. Accessed May 5, 2023.

[49] Caruana D. Heated Tobacco as Part Japan’s Successful Smoking Reduction Model. *Vaping Post*. 2021. https://www.vapingpost.com/2021/09/01/heated-tobacco-as-part-japans-successful-smoking-reduction-model/. Accessed May 5, 2023.

[50] Asia Harm Reduction Forum. *Program Rundown*. https://asiaharmreductionforum.com/pages/program_rundown_2019; Published 2019. Accessed May 5, 2023.

