# Supplementary material for: “Keep it a secret”: Leaked Documents Suggest Philip Morris International, and Its Japanese Affiliate, Continue to Exploit Science for Profit
Source: Nicotine Tob Res. 2024 Jun 27;27(5):794–804. doi: 10.1093/ntr/ntae101 (PMC12012232; doi:10.1093/ntr/ntae101)
Supplement: ntae101_suppl_Supplementary_Material [file ntae101_suppl_supplementary_material.docx]

**“Keep it a secret”: leaked documents suggest Philip Morris International, and its Japanese affiliate, continue to exploit science for profit.**

Supplementary Appendix

**REFERENCES 51-82.**

51. GFN Conference Team. 6th Global Forum on Nicotine. GFN. https://gfn.events/documents/134/gfn_2019_reader.pdf; Published 2019. Accessed 9th February 2023.

52. CoEHAR. 31 May 2022- No Tobacco Day: CoEHAR National Conference. https://web.archive.org/web/20191009172456/https:/asiaharmreductionforum.com/pages/program_rundown_2019. Published 2022. Accessed 5th May 2023.

53. SCOHRE. PROGRAMME. 4th Scientific Summit on Tobacco Harm Reduction: Novel Products, Research & Policy. https://www.nosmokesummit.org/wp-content/uploads/2021/09/2021.09.17_Programme_4th_Summit_29-30.SEPT_.pdf. Published 2021. Accessed 5th May 2023.

54. Philip Morris Products SA. Effect of Switching From Cigarette Smoking to the Use of IQOS on Periodontitis Treatment Outcome. ClinicalTrials.gov. https://www.clinicaltrials.gov/study/NCT03364751. Published 2023. Accessed 9th February 2023.

55. Yuriko S. RE: [Please confirm] 14C and 31C projects. 13 Dec 2018. Philip Morris International. https://www.industrydocuments.ucsf.edu/docs/fqcv0284. Accessed 14th June 2024.

56. Unknown. Shea Lih Goh profile. LinkedIn. https://jp.linkedin.com/in/shea-lih-goh-125a4a8. Published 2023. Accessed 9th February 2023.

57. The Dream Collective. Leading from the Living Room: PMJ. https://www.thedreamcollective.com.au/blog/leading-from-the-living-room-pmj/. Published 2021. Accessed 9th February 2023.

58. Boulton J. RE: A Question. 31 Oct 2019. Philip Morris International. https://www.industrydocuments.ucsf.edu/docs/npcv0284. Accessed 14th June 2024.

59. Unknown. A Question. 29 Oct 2019. Philip Morris International. https://www.industrydocuments.ucsf.edu/docs/xqcv0284. Accessed 14th June 2024.

60. Unknown. tomoyasu hirano. ORCID. https://orcid.org/0000-0001-8568-3856. Published 2022. Accessed 28th November 2022.

61. Unknown. Congratulations [External] FDA permits sale of IQOS Tobacco Heating System through premarket tobacco product application pathway. 07 May 2019. Philip Morris International. https://www.industrydocuments.ucsf.edu/docs/kpcv0284. Accessed 14th June 2024.

62. Hirano T, Shobayashi T, Takei T, Wakao F. Exposure Assessment of Environmental Tobacco Aerosol from Heated Tobacco Products: Nicotine and PM Exposures under Two Limited Conditions. *Int J Environ Res Public Health.* 2020;17(22).

63. Hirano T, Takei T. Estimating the Carcinogenic Potency of Second-Hand Smoke and Aerosol from Cigarettes and Heated Tobacco Products. *Int J Environ Res Public Health.* 2020;17(22).

64. Philip Morris International. The story of THS in Japan, an interview with Tomoko Iida. https://www.pmiscience.com/en/news-events/scientific-update-magazine/the-story-of-ths-in-japan--an-interview-with-tomoko-iida-/. Published 2022. Accessed 28th November 2022.

65. National Institute of Public Health. The National Institute of Public Health conducts training and research related to public health, environmental hygeine, and social welfare. https://www.niph.go.jp/en/index/. Published 2023. Accessed 7th November 2023.

66. Philip Morris International. 【前編】時代のスタンダードは燃焼から加熱へ ―燃やす・燃やさないで何が違う？―. https://www.pmj-science.com/topics/topics08/?utm_source=M3_article&utm_medium=display&utm_content=WC_no3. Published *Undated*. Accessed 9th February 2023.

67. Philip Morris Japan. サステナビリティレポート. 日本における取組みﾠ2020. https://www.pmi.com/resources/docs/default-source/japan-market/pmj_sustainability_report_2020747494be6c7468f696e2ff0400458fff.pdf?sfvrsn=e700fcb7_2. Published 2020. Accessed 9th February 2023.

68. Australian Government Department for Health. Request for Scheduling Expemtion. Philip Morris Limited. https://www.tga.gov.au/sites/default/files/foi-1482-01.pdf. Published 2019. Accessed 9th February 2023.

69. Japan Parliamentarians Federation for Population. JPFP Newsletter. https://www.apda.jp/pdf/e_newsletter/enews_no101_en.pdf. Published 2020. Accessed 7th November 2023.

70. Tobacco Tactics. Centre for Research Excellence: Indigenous Sovereignty and Smoking (COREISS). https://tobaccotactics.org/article/coreiss/. Published 2022. Accessed 5th May 2023.

71. Tobacco Tactics. The NOSMOKE Institute at Patras Science Park. https://tobaccotactics.org/article/nosmoke-institute/. Published 2022. Accessed 5th May 2023.

72. Tobacco Tactics. Global Tobacco and Nicotine Forum 2019. https://tobaccotactics.org/article/global-tobacco-and-nicotine-forum-2019/. Published 2020. Accessed 5th May 2023.

73. Velicer C, St Helen G, Glantz SA. Tobacco papers and tobacco industry ties in regulatory toxicology and pharmacology. *J Public Health Policy.* 2018;39(1):34-48.

74. Chapman S. Philip Morris speaks at and promotes an obscure conference on lung disease. *The Conversation.* 2016. https://theconversation.com/philip-morris-speaks-at-and-promotes-an-obscure-conference-on-lung-disease-62497. Accessed 28th November 2022.

75. Queloz S, Etter J-F. An online survey of users of tobacco vaporizers, reasons and modes of utilization, perceived advantages and perceived risks. *BMC Public Health.* 2019;19(1):642.

76. Sutanto E, Miller C, Smith DM, et al. Prevalence, Use Behaviors, and Preferences among Users of Heated Tobacco Products: Findings from the 2018 ITC Japan Survey. *Int J Environ Res Public Health.* 2019;16(23):4630.

77. Tompkins CNE, Burnley A, McNeill A, Hitchman SC. Factors that influence smokers’ and ex-smokers’ use of IQOS: a qualitative study of IQOS users and ex-users in the UK. *Tob Control.* 2020:tobaccocontrol-2019-055306.

78. Philip Morris International. Do heated tobacco products really contribute to harm reduction for public health? https://www.pmiscience.com/en/news-events/news/heated-tobacco-products-public-health-harm-reduction/. Published 2023. Accessed 7th November 2023.

79. Godlee F, Malone R, Timmis A, et al. Journal policy on research funded by the tobacco industry. *The BMJ.* 2013;347:f5193.

80. Kojima T, Green J, Barron JP. Conflict-of-interest disclosure at medical journals in Japan: a nationwide survey of the practices of journal secretariats *BMJ Open.* 2015;5: e007957.

81. Branston JR. Industry profits continue to drive the tobacco epidemic: A new endgame for tobacco control? *Tob Prev Cessat.* 2021;7(June):1-3.

82. Alomar D. Tobacco majors spent billions on R&D of reduced-risk alternatives to smoking since 2008, says exec. *Arab News.* 2022. https://www.arabnews.com/node/2127701/business-economy. Accessed February 2023.

83. Cohen JE, Zeller M, Eissenberg T, et al. Criteria for evaluating tobacco control research funding programs and their application to models that include financial support from the tobacco industry. *Tob Control.* 2009;18(3):228-234.

84. Philip Morris Products S.A. Comparison of Abdominal Aortic Aneurysm Growth in Adult Smoking Patients Who Either Switch to IQOS, Continue Smoking, or Quit Smoking. ClinicalTrials.gov. https://www.clinicaltrials.gov/study/NCT03837704. Published 2023. Accessed 30th June 2023.

85. The Federal Department of Foreign Affairs. About us. https://houseofswitzerland.org/about-us. Published 2022. Accessed 28th November 2022.
